# Benefit of Early Ruxolitinib Initiation Regardless of Fibrosis Grade in Patients with Primary Myelofibrosis: A Post Hoc Analysis of the Single-Arm Phase 3b JUMP Study

**DOI:** 10.3390/cancers15102859

**Published:** 2023-05-22

**Authors:** Francesca Palandri, Haifa Kathrin Al-Ali, Paola Guglielmelli, Mike W. Zuurman, Rajendra Sarkar, Vikas Gupta

**Affiliations:** 1 Istituto di Ematologia “Seràgnoli”, IRCCS Azienda Ospedaliero-Universitaria di Bologna, 40138 Bologna, Italy; 2 University Hospital Halle, 06120 Halle (Saale), Germany; 3 Center of Research and Innovation of Myeloproliferative Neoplasms, Azienda Ospedaliera-Universitaria Careggi, University of Florence, 50134 Florence, Italy; 4 Novartis Pharma AG, 4056 Basel, Switzerland; 5 Novartis Healthcare Private Limited, Hyderabad 500081, India; 6 Princess Margaret Cancer Centre, Toronto, ON M5G 2C4, Canada

**Keywords:** primary myelofibrosis, ruxolitinib, bone marrow fibrosis, spleen response, phase 3b trial, JAK1/2 inhibition

## Abstract

**Simple Summary:**

Bone marrow fibrosis (BMF) is a hallmark of myelofibrosis (a rare blood cancer), and BMF severity may be useful in predicting patient response to treatment, since high-severity BMF is associated with poor survival. In this study, we studied how BMF severity and early (within 2 years of diagnosis) versus late (more than 2 years after diagnosis) initiation of ruxolitinib treatment affected the response to treatment in patients with primary myelofibrosis. Our results showed that patients with low-severity BMF had a better response to treatment with ruxolitinib and longer survival; however, all patients treated with ruxolitinib showed improvements in spleen size and survival, regardless of their BMF severity. Our data also showed that initiation of early treatment with ruxolitinib resulted in better responses in all patients. This study showed that treatment with ruxolitinib can benefit patients with both low- and high-severity BMF, especially when it is started early.

**Abstract:**

Bone marrow fibrosis (BMF) is an adverse prognostic factor for myelofibrosis (MF). The single-arm, open-label, phase 3b JUMP trial (NCT01493414) assessed the safety and efficacy of the JAK1/JAK2 inhibitor ruxolitinib in patients with symptomatic MF. This post hoc analysis investigated the impact of BMF grade on response and outcomes in patients with primary MF (PMF) from the JUMP study. BMF was assessed by biopsy and graded from 0 to 3; grades 0–1 were considered low-grade fibrosis (LGF) and grades 2–3 were considered high-grade fibrosis (HGF). Patients with LGF (n = 268) had lower rates of cytopenias at baseline but showed comparable disease burden vs. patients with HGF (n = 852). The proportion of patients achieving a spleen response was greater in the LGF group vs. the HGF group at Week 24 and at any time during the study, while overall survival estimates were improved in patients with LGF vs. patients with HGF. Early initiation of ruxolitinib therapy (within 2 years of diagnosis) was associated with increased response rates in all patients. These results highlight the efficacy of ruxolitinib in symptomatic patients with PMF, with the greatest clinical improvements observed in patients with LGF and in patients who received early treatment.

## 1. Introduction

Myelofibrosis (MF) is a chronic myeloproliferative neoplasm associated with cytopenias, splenomegaly, and constitutional symptoms such as weight loss, night sweats, and fever [1,2,3]. It can present as a primary disorder, known as primary MF (PMF), or secondary to polycythemia vera (PV) or essential thrombocythemia (ET) [1,2,3]. These are rare disorders, with reported annual incidence rates from 0.01 to 2.61, 0.21 to 2.27, and 0.22 to 0.99 per 100,000 for PV, ET, and PMF, respectively [4]. MF is a progressive disease, with significantly more patients experiencing anemia, thrombocytopenia, constitutional symptoms, and splenomegaly 1 year after diagnosis compared with patients evaluated at the time of diagnosis [5]. 

Bone marrow fibrosis (BMF) is one of the main characteristics of MF [3,6]. Fibrosis, angiogenesis, and osteosclerosis are commonly found during bone marrow histology of patients with PMF, whereas advanced reticulin and collagen fibrosis are not always present [7]. The pathogenesis of BMF is not well understood but appears to derive from cytokine stimulation of fibroblasts in bone marrow tissue by malignant hematopoietic cells [7,8]. High-grade fibrosis is a prognostic risk factor incorporated into recent risk stratification systems [7,9]; it is associated with poor prognosis of MF and is an important disease feature associated with increased morbidity and mortality in patients with PMF. 

Hyperactivation of the JAK/STAT signaling pathway is a hallmark of MF, usually as a result of mutations in the *JAK2*, *MPL*, or *CALR* genes [3,10]. Ruxolitinib is a potent and selective JAK1/JAK2 inhibitor that has been approved for the treatment of MF based on its superior efficacy vs. placebo or best available therapy in the phase 3 COMFORT I and II trials, respectively [11,12]. In these trials, treatment with ruxolitinib led to reductions in spleen size, improvements in symptoms and quality of life (QoL) assessments, and longer overall survival (OS) [11,12,13,14].

The single-arm, open-label, phase 3b, expanded-access JAK Inhibitor rUxolitinib in Myelofibrosis Patients (JUMP) trial (ClinicalTrials.gov NCT01493414) assessed the safety and efficacy of ruxolitinib in patients with symptomatic MF without access to ruxolitinib outside of the clinical trial setting [15]. A total of 2233 patients were enrolled, making JUMP the largest and most expansive clinical trial in patients with MF treated with ruxolitinib to date. Meaningful reductions in spleen length and symptom improvement were observed in patients treated with ruxolitinib in a setting that was comparable to routine clinical practice, confirming the results of previous studies. The safety profile of ruxolitinib in JUMP was consistent with that observed in previous reports [15], with no new safety concerns identified.

This post hoc analysis of the JUMP study was conducted to evaluate the impact of BMF grade and timing of treatment initiation on response and outcomes in patients with PMF who were treated with ruxolitinib.

## 2. Materials and Methods

### 2.1. Study Design

JUMP was a single-arm, multicenter, phase 3b study of ruxolitinib in patients with MF who were unable to access ruxolitinib outside of a clinical trial setting. This expanded-access study was designed to collect additional safety and efficacy data on ruxolitinib while also providing an access pathway for patients. The post hoc analysis described here stratified patients by BMF grade into a low-grade fibrosis (LGF) group (Grade 0 or Grade 1 BMF) and a high-grade fibrosis (HGF) group (Grade 2 or Grade 3 BMF). Patients were also stratified for certain analyses by time since diagnosis and by time since last biopsy. 

The JUMP study design has been described previously [15]. Patients were treated at clinical sites across 26 countries, including countries in Europe, Latin America, and North America, between August 2011 and January 2017. Eligible patients were ≥18 years of age and diagnosed with PMF or secondary MF according to the 2008 revised WHO criteria [16,17]. Patients with low platelet counts and patients without splenomegaly—patient populations that have not been extensively studied—were both included in the trial. Patients eligible for hematopoietic stem cell transplantation and patients with a history of malignancy in the previous 3 years were excluded from the study. Starting doses of ruxolitinib were based on platelet counts at baseline: 5 mg twice daily (bid; 50–<100 × 10^9^/L), 15 mg bid (100–200 × 10^9^/L), or 20 mg bid (>200 × 10^9^/L). Ruxolitinib dose was titrated for each patient (up to a maximum of 25 mg bid) and patients were treated for up to 24 months after the last patient’s first visit (23 December 2014), until disease progression, unacceptable toxicity, death, discontinuation from the study for any other reason, or if the drug became commercially available. Patients were followed up for 28 days after the end-of-treatment visit; no data were collected for patients beyond this visit, including those who continued treatment with commercially available ruxolitinib (Supplementary Figure S1). 

### 2.2. Endpoints

The primary endpoint of this study was safety and tolerability of ruxolitinib (as assessed by the incidence and severity of adverse events [AEs]). Secondary endpoints included spleen response (as assessed by reduction in spleen length from baseline and expressed as the proportion of patients with a ≥50% reduction in palpable spleen length); patient-reported outcomes (Functional Assessment of Cancer Therapy–Lymphoma total score [FACT-Lymphoma total score] and Functional Assessment of Chronic Illness Therapy [FACIT]–Fatigue scale); progression-free survival (PFS, defined as time from first study drug administration to date of documented progression based on the International Working Group for Myelofibrosis Research and Treatment [IWG-MRT] response criteria); and OS. The IWG-MRT criteria for progression include the appearance of new splenomegaly that is palpable at least 5 cm below the left costal margin (LCM); a ≥100% increase in palpable distance, below LCM, for baseline splenomegaly of 5–10 cm; a 50% increase in palpable distance, below LCM, for baseline splenomegaly of >10 cm; leukemic transformation confirmed by a bone marrow blast count of ≥20% or a peripheral blood blast content of ≥20% associated with an absolute blast count of ≥1 × 10^9^/L that lasts for at least 2 weeks [18]. Changes in spleen length were assessed by manual palpation with spleen response evaluated according to the IWG-MRT criteria; spleen response was assessed in patients with baseline and post-baseline measurements. Patients with missing data on spleen length at baseline or at a post-baseline visit were excluded from the analysis. PFS and OS were assessed using the Kaplan-Meier method. Fibrosis grade was assessed in hematopoietic areas of the bone marrow as Grades 0–3 in patients with available bone marrow biopsy results [19].

This post hoc analysis was not statistically powered, and all results are of a descriptive nature.

### 2.3. Ethics

The study was conducted in line with the Declaration of Helsinki, and written informed consent was obtained from each patient prior to the initiation of study screening activities. The study protocol and all amendments were reviewed by the Independent Ethics Committee or the Institutional Review Board for each center. The study was funded by Novartis Pharmaceuticals Corporation and is registered with ClinicalTrials.gov (NCT01493414). 

## 3. Results

### 3.1. Baseline Characteristics

A total of 1326 patients with PMF were enrolled in the JUMP study; of these, 1,120 had a documented biopsy with BMF grade assessment (Grade 0, n = 25; Grade 1, n = 243; Grade 2, n = 433; Grade 3, n = 419; missing, n = 206). Patients were stratified according to their BMF grade into LGF (Grades 0–1, n = 268) or HGF (Grades 2–3, n = 852) groups. The median (range) daily dose of ruxolitinib was similar for patients with LGF (30 mg [4–50]) and patients with HGF (29 mg [6–50]). Median (range) duration of exposure was also comparable between LGF (17 months [0–59]) and HGF (13 months [0–60]) groups.

Baseline characteristics were similar between patients with LGF and HGF (Table 1). There were more patients aged 65 years or older in the HGF group. A larger proportion of patients with LGF had low or intermediate-1 Dynamic International Prognostic Scoring System (DIPSS) scores [20,21] compared with patients with HGF. Larger numbers of patients with HGF had anemia and thrombocytopenia at baseline, as shown by hemoglobin levels, the proportion of patients receiving transfusions, and platelet counts. However, both LGF and HGF groups experienced comparable PMF symptom burdens, as shown by the similar proportions of patients with splenomegaly, spleen length, and QoL scores at baseline. 

### 3.2. Patient Disposition

Treatment completion rates for patients with LGF and HGF were similar (55.6% vs. 54.0%), with AEs being the primary reason for treatment discontinuation in both groups (LGF, 23.5%; HGF, 18.1%). Few patients discontinued treatment due to disease progression (9.0% vs. 10.2%) or death (4.9% vs. 5.6%) in both groups (Supplementary Figure S2).

### 3.3. Spleen Response

**Figure 1:**
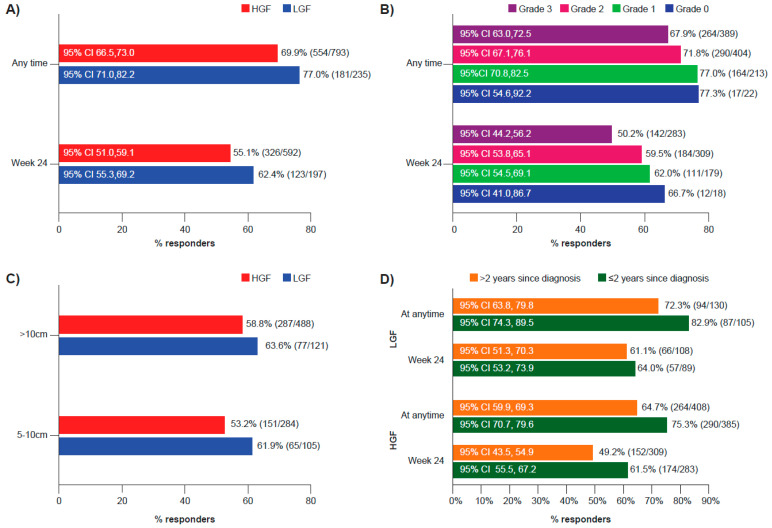
Spleen response in patients with PMF based on fibrosis grade. (**A**) Proportion of patients with ≥50% spleen length reduction from baseline at Week 24 and at any time during the study stratified by LGF or HGF. (**B**) Proportion of patients with ≥50% spleen length reduction from baseline at Week 24 and at any time during the study stratified by fibrosis grade (0, 1, 2, and 3). (**C**) Proportion of patients with ≥50% spleen length reduction as the best overall response based on the IWG-MRT criteria during the study stratified by spleen size at baseline. Patients with spleen lengths less than 5 cm were not evaluable for response. (**D**) Proportion of patients with ≥50% spleen length reduction from baseline at Week 24 and at any time during the study stratified by LGF or HGF and time to initiation of ruxolitinib therapy post-diagnosis. The best overall response to treatment was assessed by spleen palpation (calculated as the percentage change in spleen length compared with baseline) unless otherwise noted. CI, confidence interval; HGF, high-grade fibrosis; IWG-MRT, International Working Group for Myelofibrosis Research and Treatment; LGF, low-grade fibrosis; PMF, primary myelofibrosis.

The proportion of patients achieving a spleen response was greater in patients with LGF compared with patients with HGF. This was evident at both Week 24 and at any time during the study (Figure 1A). A clear trend between fibrosis grade and spleen response rate was observed for patients achieving a spleen response at Week 24 or at any time during the study, with response rates decreasing with increasing fibrosis grade (Figure 1B). The increased proportion of patients with LGF achieving a spleen response was independent of spleen size at baseline (Figure 1C). Early initiation of ruxolitinib therapy (within 2 years of diagnosis) was associated with increased response rates in both LGF and HGF groups (Figure 1D). A similar trend was observed for response rates in patients starting ruxolitinib therapy within 1 year of diagnosis (87.0% [67/77] of responders in patients with LGF and 78.1% [218/279] in patients with HGF) vs. those who started ruxolitinib 1 year after diagnosis (72.2% [114/158] of responders in patients with LGF and 65.4% [336/514] in patients with HGF). 

In patients with PMF, BMF can increase as the disease progresses [6,22]; therefore, patients who were initially classified as having LGF may have progressed to higher-grade fibrosis over time. A sub-analysis was thus carried out stratifying patients by time since last biopsy in order to highlight any potential discrepancies in efficacy data in patients for whom a longer period of time had elapsed between their last biopsy and ruxolitinib treatment initiation. As shown in Supplementary Table S1, patients in the LGF group had consistently higher spleen response rates than patients in the HGF group at both Week 24 and at any time during the study, irrespective of the time since their last biopsy. Stratifying patients by time since diagnosis and time since last biopsy showed that spleen response rates at Week 24 were not substantially different for patients with LGF who started ruxolitinib within 2 years of diagnosis and those who started later. However, for patients with HGF, the best results in terms of spleen response were obtained when ruxolitinib treatment was started within 2 years of both diagnosis and time of latest biopsy (Supplementary Figure S3).

### 3.4. Progression-Free Survival

Median (range) study follow-up was 18.0 (0.7–59.8) months for patients with LGF and 14.3 (<0.1–60.6) months for patients with HGF. At Week 144, PFS estimates (95% CI) were greater for patients with LGF (82% [0.76, 0.88]) than for patients with HGF (70% [0.65, 0.74]). Death and progression events occurred in 14.6% of patients with LGF vs. 18.3% of patients with HGF.

PFS estimates were higher for patients with LGF who initiated ruxolitinib treatment within 2 years of their last biopsy vs. patients with HGF; however, there were no clear PFS differences between patients with LGF and patients with HGF who started treatment with ruxolitinib more than 2 years after their last biopsy (Supplementary Figure S4).

### 3.5. Overall Survival

**Figure 2:**
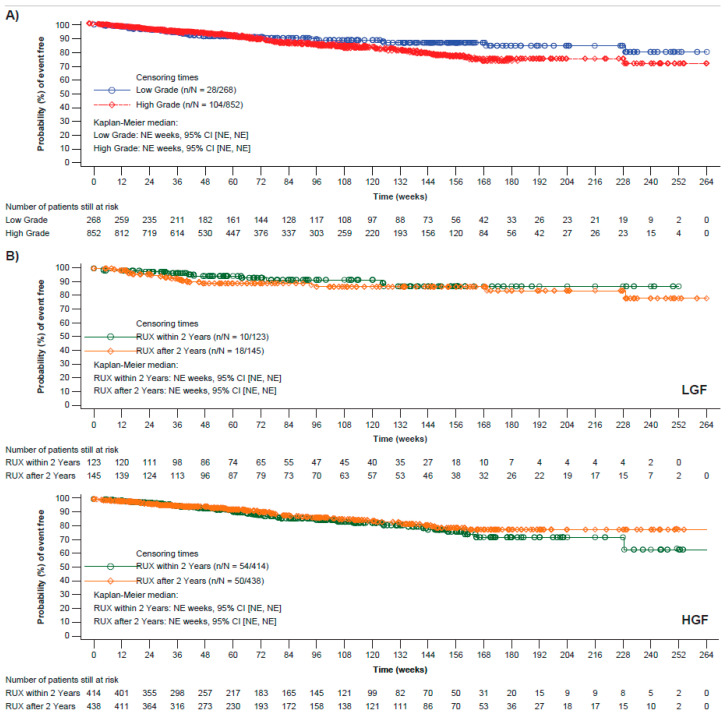
OS in patients with PMF based on fibrosis grade. (**A**) OS estimates in patients with LGF and HGF. (**B**) OS estimates in patients with LGF (**top panel**) and HGF (**lower panel**) stratified by time of ruxolitinib treatment initiation. CI, confidence interval; HGF, high-grade fibrosis; LGF, low-grade fibrosis; NE, not estimable; OS; overall survival; PMF, primary myelofibrosis; RUX, ruxolitinib.

Patients with LGF had improved OS (Figure 2A), with estimates (95% CI) at Week 144 of 87% (0.81, 0.91) vs. 79% (0.75, 0.83) for patients with HGF. Lower fibrosis grades correlated with better OS estimates (95% CI) at Week 144 (Grade 0, 91% [0.68, 0.98]; Grade 1, 87% [0.80, 0.91]; Grade 2, 80% [0.74, 0.85]; and Grade 3, 78%, [0.72, 0.84]). Deaths were reported for 8.0% of patients with Grade 0 fibrosis; 10.7% of patients with Grade 1 fibrosis; 11.3% of patients with Grade 2 fibrosis; and 13.1% of patients with Grade 3 fibrosis. A trend of improved OS with early initiation of ruxolitinib treatment was noted in patients with LGF, but not in patients with HGF (Figure 2B). Deaths were reported for 8.1% of patients with LGF who had initiated ruxolitinib treatment within 2 years of diagnosis vs. 12.4% for those who had initiated ruxolitinib 2 years after their diagnosis. Similar results were observed when ruxolitinib was initiated within 1 year of diagnosis.

OS estimates were slightly higher for patients with LGF initiating ruxolitinib treatment within 2 years of their last biopsy vs. patients with HGF, and similar for patients who started treatment with ruxolitinib more than 2 years after their last biopsy regardless of BMF grade (Supplementary Figure S5).

### 3.6. Safety

The incidences of hematological AEs were similar for patients in both LGF and HGF groups (Table 2). However, patients with HGF experienced Grade 3/4 AEs more frequently than patients with LGF (48.1% vs. 39.2%). While the proportion of patients with transfusion dependency decreased over time in both groups, the decrease was larger for patients with LGF (27.3% of patients with LGF who were transfusion-dependent at baseline became transfusion-independent at the end of the study vs. 18.2% of HGF patients).

## 4. Discussion

This post hoc analysis of patients with PMF enrolled in the JUMP study—the largest cohort of patients treated with ruxolitinib to date—highlights the efficacy and safety of ruxolitinib for this patient population. All patients, regardless of BMF grade, experienced clinically meaningful improvements in spleen response, PFS, and OS, with a manageable safety profile. Although improvements in spleen responses were achieved in both LGF and HGF patient groups, these were consistently greater in patients with LGF, despite overlapping confidence intervals. The high symptom burden, splenomegaly, and poor QoL scores at baseline for patients with LGF indicate that these patients experience a disease comparable in severity and impact on daily life to that of patients with HGF.

Patients with HGF were more likely to present with anemia and/or thrombocytopenia at baseline and were also more likely to have received prior transfusions. However, patients with LGF and HGF experienced similar symptomatology, comparable rates and severity of splenomegaly, and similar QoL scores at baseline, which may be explained by the fact that this study only included patients for whom treatment with ruxolitinib was deemed appropriate. This is in agreement with results from the MPN Landmark survey, which showed that patients with MF and low symptom severity reported that their disease-related symptoms reduced their QoL, impairing their ability to work and attend social events [23,24]. Overall, these results suggest that patients with LGF experience a high disease burden and underscore their unmet medical needs.

Patients with LGF and HGF obtained substantial clinical benefits from ruxolitinib treatment, as evidenced by spleen response, OS, and PFS data. However, patients with LGF tended to derive greater benefits from ruxolitinib than patients with HGF. This is in agreement with a previous study showing that increasing BMF grade correlates with poorer survival [25]. Ruxolitinib treatment has been associated with a delay of BMF progression, as well as BMF improvement or stabilization in patients enrolled in the COMFORT studies [11,26]; this suggests that long-term treatment with ruxolitinib increases the chances of achieving spleen responses and improves PFS and OS in patients with PMF.

Spleen response rates in this study were improved by early initiation of ruxolitinib treatment. Although the proportion of patients achieving a spleen response was consistently higher in patients with LGF, patients with HGF also benefited from early treatment initiation, with higher response rates in patients who started ruxolitinib within 2 years of diagnosis. The best spleen responses at Week 24 for patients with HGF were observed for patients who started treatment with ruxolitinib within two years of their diagnosis and last biopsy, illustrating that patients with delayed treatment initiation may experience poorer outcomes. Given that MF is a progressive disease [5], treating patients early after diagnosis may contribute to improved outcomes. Indeed, first-line treatment with ruxolitinib was an independent predictor of spleen response in the JUMP study [27]. A reduction in spleen size at Week 24 correlated with longer survival in patients enrolled in COMFORT I and II studies [28] and previous analyses of long-term survival in patients from these studies have shown that earlier treatment with ruxolitinib (≤2 years vs. >2 years from diagnosis) led to higher rates of spleen and symptom responses, as well as longer OS [29]. Furthermore, an independent Italian study of 408 patients with MF found that initiating ruxolitinib treatment more than 2 years after diagnosis correlated negatively with spleen response [30]. Overall, these results highlight the importance of starting treatment with ruxolitinib early after disease diagnosis in order to maximize benefits.

The high proportion of patients with LGF and HGF completing treatment as per the protocol underscores the efficacy and safety of ruxolitinib for this patient population, with few patients discontinuing due to disease progression, AEs, or death. Based on the mechanism of action of ruxolitinib, anemia and thrombocytopenia are expected and common AEs, but are usually transient, manageable, and rarely lead to discontinuation [11,12,15]; low rates of non-hematologic AEs have been observed with ruxolitinib treatment, primarily Grade 1/2 [11,12]. The incidences of hematological AEs were similar between patients with LGF and HGF; however, Grade 3/4 AEs occurred more frequently in patients with HGF. These results were consistent with previous analyses of patients in the JUMP study who were stratified by dynamic IPSS (DIPSS), with high-risk patients experiencing significantly greater rates of Grade ≥3 AEs [31,32]. While BMF is not included in DIPSS scoring, greater BMF is indicative of more advanced disease in patients with PMF. A possible limitation of this analysis is that LGF could be a marker associated with prognostically favorable clinical characteristics in patients with PMF, as shown by the smaller proportion of patients with cytopenia at baseline. It is worth noting that the group of patients with HGF included an increased proportion of older patients (aged 65 or older), as well as increased proportions of patients with intermediate-2 and high IPSS risks; age and high-risk IPSS are both markers of poor prognosis, which could in part explain our results [20]. In addition, there was no central review of BMF, and fibrosis data were captured by the participating center in the case report forms via predefined fields. Due to the JUMP study design and intent, it is important to note that this post hoc analysis was designed to test any hypothesis, therefore all results presented are descriptive.

Ruxolitinib was the first JAK1/2 inhibitor specifically approved for the treatment of MF and was followed by the approval of fedratinib for patients with resistance or intolerance to ruxolitinib [33,34]. More recently, pacritinib (a JAK2 inhibitor) was approved for patients with intermediate- or high-risk MF and severe thrombocytopenia (platelet count <50 × 10^9^/L) [35]. However, anemia remains a major challenge and a significant clinically unmet need in patients with MF. Momelotinib, a JAK1/JAK2 inhibitor with additional activity against ACVR1, is in late-stage clinical development and is hoped to address the unmet need of anemia in patients with MF [36]. Furthermore, the simultaneous targeting of multiple pathways may offer a new therapeutic strategy to maximize the efficacy of JAK inhibition, particularly in patients with a loss of response to JAK inhibitors. Promising clinical outcomes were observed in patients with MF who were treated with ruxolitinib in combination with parsaclisib (PI3Kδ inhibitor), umbralisib (PI3Kδ inhibitor), pelabresib (BET inhibitor), and navitoclax (BCL-X_1_/BCL-2 inhibitor) [37,38,39,40]. Preliminary data also suggest that an investigation of the use of TP-3654 in combination with ruxolitinib is warranted [41].

## 5. Conclusions

Patients with PMF and LGF experienced higher response rates and had improved OS estimates compared with patients with HGF. A greater clinical benefit (as evidenced by improvements in spleen response and OS) was observed in patients with PMF who initiated ruxolitinib within 2 years of diagnosis compared with those who initiated treatment later, regardless of BMF grade, underscoring the importance of early initiation of treatment with ruxolitinib in these patients.

## Figures and Tables

**Table 1:** Patient baseline characteristics.

	LGF N = 268	HGF N = 852
Median age (range), years	66.0 (26.0–88.0)	67.0 (18.0–89.0)
≥65 years, n (%)	143 (53.4)	536 (62.9)
Male, n (%)	164 (61.2)	503 (59.0)
Mean time since initial diagnosis (SD), months	49.1 (54.9)	47.9 (58.4)
Mean time since last biopsy (SD), months	35.5 (45.7)	26.1 (36.0)
Dynamic IPSS risk group at study entry, n (%)		
Low risk	14 (5.2)	14 (1.6)
Intermediate risk 1	112 (41.8)	290 (34.0)
Intermediate risk 2	71 (26.5)	337 (39.6)
High risk	15 (5.6)	87 (10.2)
Missing	56 (20.9)	124 (14.6)
Hemoglobin level < 100 g/L, n (%)	87 (32.5)	395 (46.4)
Platelets < 100 × 10^9^/L, n (%)	11 (4.1)	73 (8.6)
Peripheral blasts ≥ 1%, n (%)	65 (24.3)	279 (32.7)
Palpable spleen, n (%)	239 (89.2)	807 (94.7)
Mean palpable spleen length below costal margin (SD), cm	11.3 (7.1)	12.9 (7.2)
Spleen length, n (%)		
<5 cm	34 (12.7)	65 (7.6)
5–10 cm	105 (39.2)	284 (33.3)
>10 cm	121 (45.1)	488 (57.3)
Missing	8 (3.0)	15 (1.8)
Prior transfusions, n (%)	60 (22.4)	261 (30.6)
Mean FACIT–Fatigue total score (SD) ^a^	34.5 (10.8)	32.8 (12.0)
Mean FACT–Lymphoma total score (SD) ^a^	115.4 (22.0)	114.9 (23.9)

^a^ For patients with LGF (n = 241) and patients with HGF (n = 790) with an assessment. FACIT, Functional Assessment of Chronic Illness Therapy; FACT, Functional Assessment of Cancer Therapy; HGF, high-grade fibrosis; IPSS, International Prognostic Scoring System; LGF, low-grade fibrosis; SD, standard deviation.

**Table 2:** Incidence of hematological AEs in patients with PMF and LGF or HGF.

	LGF		HGF	
	n (%)		n (%)	
	N = 268		N = 852	
	All grades	Grade 3/4	All grades	Grade 3/4
Total	205 (76.5)	105 (39.2)	632 (74.2)	410 (48.1)
Anemia	171 (63.8)	90 (33.6)	489 (57.4)	334 (39.2)
Thrombocytopenia	112 (41.8)	36 (13.4)	397 (46.6)	164 (19.2)
Infection	1 (0.4)	1 (0.4)	4 (0.5)	1 (0.1)

AE, adverse event; HGF, high-grade fibrosis; LGF, low-grade fibrosis; PMF, primary myelofibrosis.

## Data Availability

Novartis is committed to sharing with qualified external researchers, access to patient-level data and supporting clinical documents from eligible studies. These requests are reviewed and approved by an independent review panel on the basis of scientific merit. All data provided are anonymized to respect the privacy of patients who have participated in the trial in line with applicable laws and regulations. Trial data availability is according to the criteria and process described at www.clinicalstudydatarequest.com (accessed on 16 May 2023).

## Supplementary Materials

The following supporting information can be downloaded at https://www.mdpi.com/article/10.3390/cancers15102859/s1, Figure S1: JUMP study design, Figure S2: Patient disposition, Figure S3: Proportion of patients with PMF achieving a spleen response at Week 24 and at any time during the study stratified by time since diagnosis and time since last biopsy, Figure S4: Progression-free survival in patients with PMF stratified by time since last biopsy, Figure S5: Overall survival in patients with PMF stratified by time since last biopsy, Table S1: Number of patients with PMF achieving a spleen response at Week 24 stratified by time since last biopsy; File S1. List of IRB-IECs_JUMP manuscript.
