# Supplementary material for: Benefit of Early Ruxolitinib Initiation Regardless of Fibrosis Grade in Patients with Primary Myelofibrosis: A Post Hoc Analysis of the Single-Arm Phase 3b JUMP Study
Source: Cancers (Basel). 2023 May 22;15(10):2859. doi: 10.3390/cancers15102859 (PMC10216208; doi:10.3390/cancers15102859)
Supplement: Supplementary file 1 [file cancers-15-02859-s001.zip › cancers-2282491-supplementary.pdf]

Supplementary materials

Supplementary Table S1. Number of patients with PMF achieving a spleen response at Week 24 stratified by time since last biopsy.

	**LGF**<br>**n (%) [95% CI]**<br>**N=113**	**HGF**<br>**n (%) [95% CI]**<br>**N=384**
**Week 24**		
Ruxolitinib initiated ≤2 years since last biopsy	72 (63.7) [54.1, 72.6]	230 (59.9) [54.8, 64.8]
Ruxolitinib initiated >2 years since last biopsy	51 (60.7) [49.5, 71.2]	96 (46.2) [39.2, 53.2]
**At any time during the study**		
Ruxolitinib initiated ≤2 years since last biopsy	109 (81.3) [73.7, 87.5]	382 (73.0) [69.0, 76.8]
Ruxolitinib initiated >2 years since last biopsy	72 (71.3) [61.4, 79.9]	172 (63.7) [57.7, 69.4]

Spleen response was assessed by reduction in spleen length from baseline and expressed as the proportion of patients with a ≥50% reduction in palpable spleen length. CI, confidence interval; HGF, high-grade fibrosis; LGF, low-grade fibrosis; PMF, primary myelofibrosis.

Supplementary Figure S1. JUMP study design.

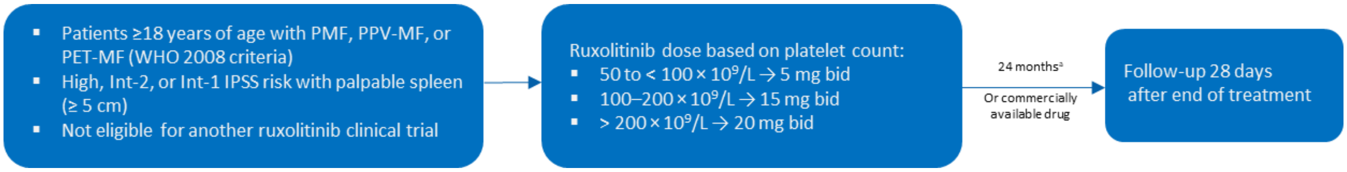

[a] Patients could also discontinue treatment in the event disease progression, unacceptable toxicity, death, or discontinuation from the study for any other reason.

bid, twice daily; Int, intermediate; IPSS, International Prognostic Scoring System; PET-MF, post-essential thrombocythemia myelofibrosis; PMF, primary myelofibrosis; PPV-MF, post-polycythemia vera myelofibrosis.

Supplementary Figure S2. Patient disposition.

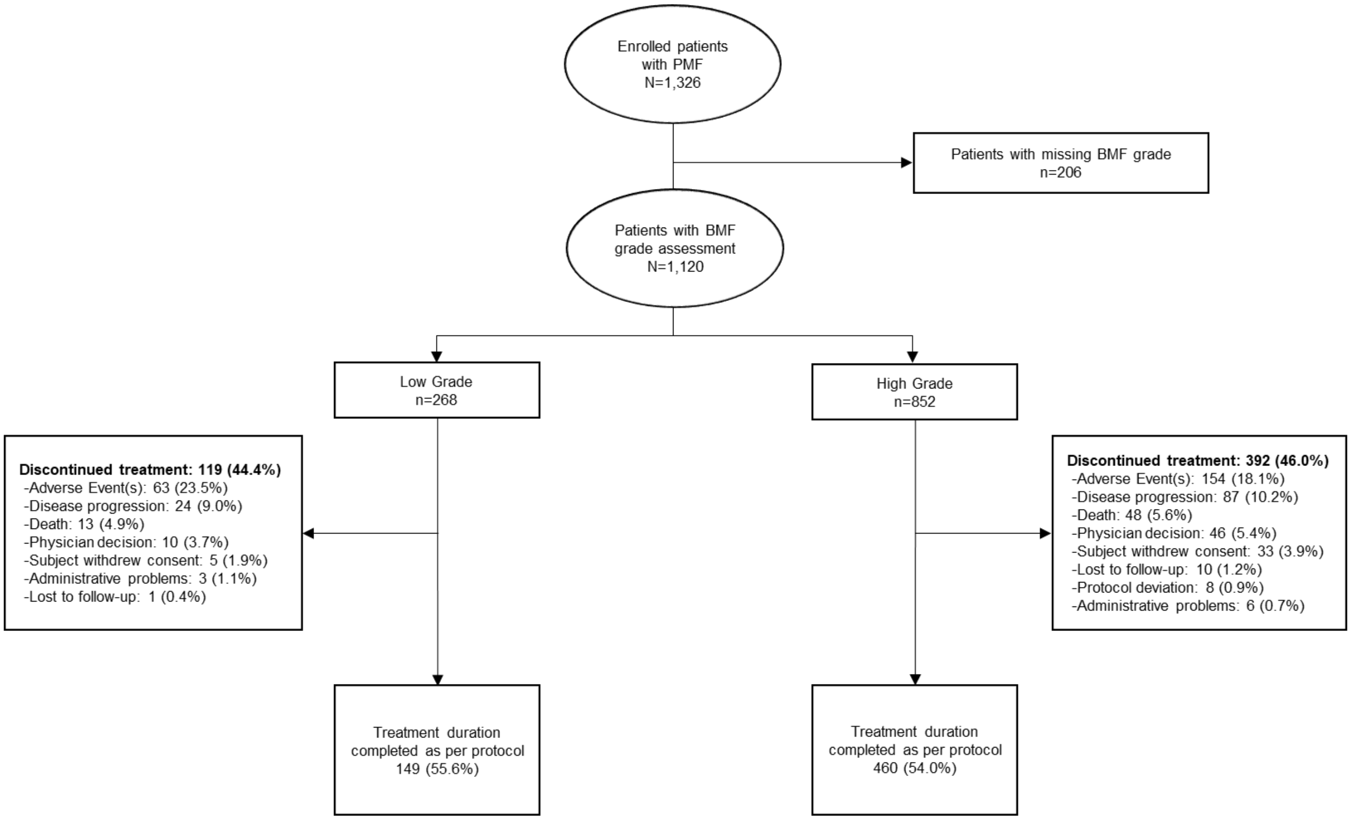

BMF, bone marrow fibrosis; PMF, primary myelofibrosis.

Supplementary Figure S3. A. Proportion of patients with PMF achieving a spleen response at Week 24 stratified by time since diagnosis and time since last biopsy. **B.** Proportion of patients with PMF achieving a spleen response at any time during the study stratified by time since diagnosis and time since last biopsy.

A)

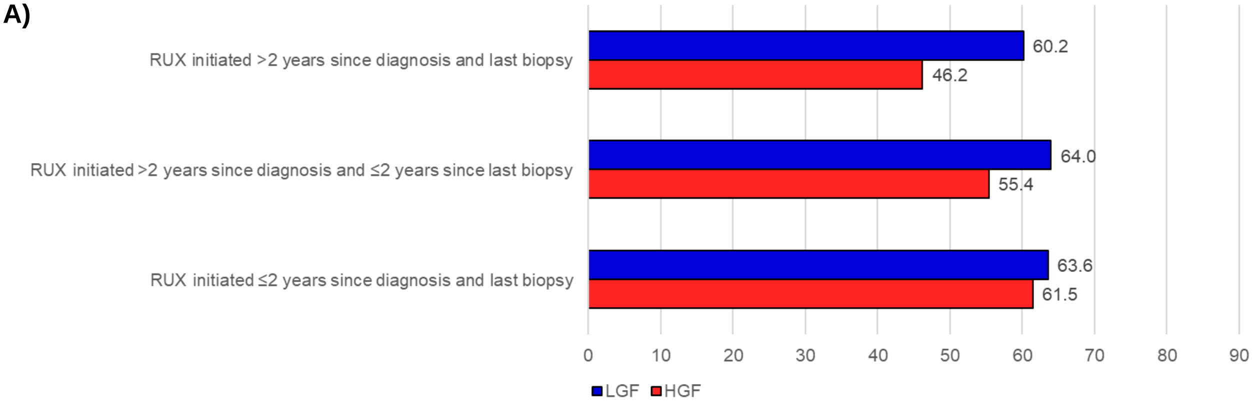

B)

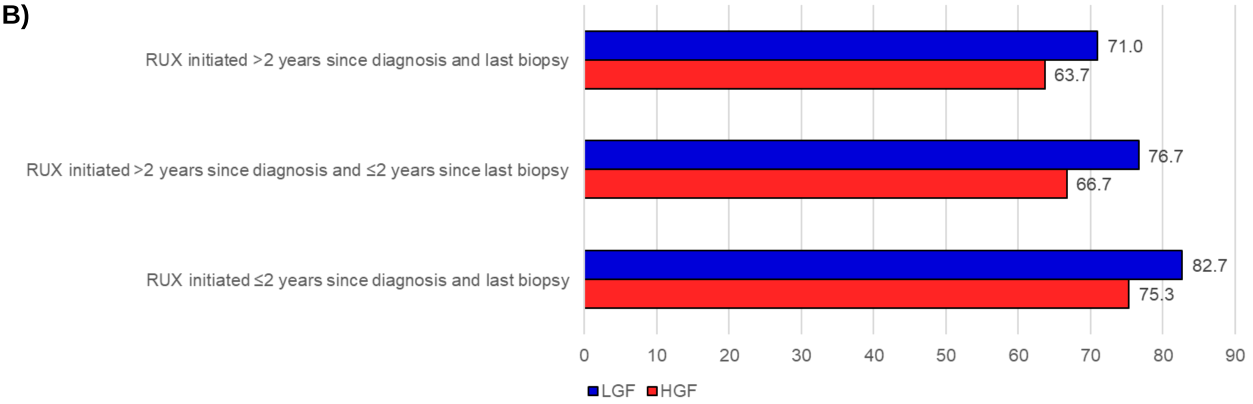

HGF, high-grade fibrosis; LGF, low-grade fibrosis; PMF, primary myelofibrosis; RUX, ruxolitinib.

Supplementary Figure S4. Progression-free survival in patients with PMF stratified by time since last biopsy. **A**, Patients who started ruxolitinib treatment ≤2 years since their last biopsy. **B**, Patients who started ruxolitinib treatment >2 years since their last biopsy.

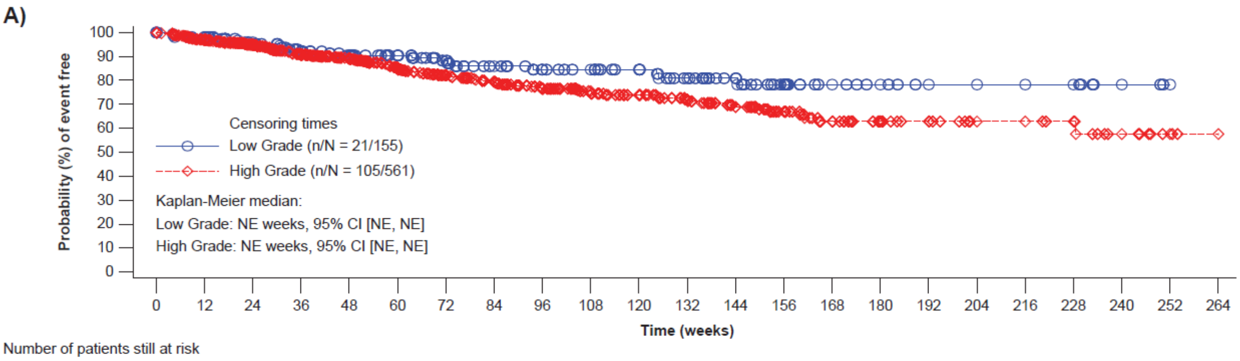

Number of patients still at risk	0	12	24	36	48	60	72	84	96	108	120	132	144	156	168	180	192	204	216	228	240	252	264
Low Grade	155	149	133	116	105	91	77	66	59	55	49	40	32	22	12	9	6	6	6	6	2	0	
High Grade	561	516	446	375	319	264	223	200	172	145	125	104	83	60	41	28	22	15	15	13	7	2	0

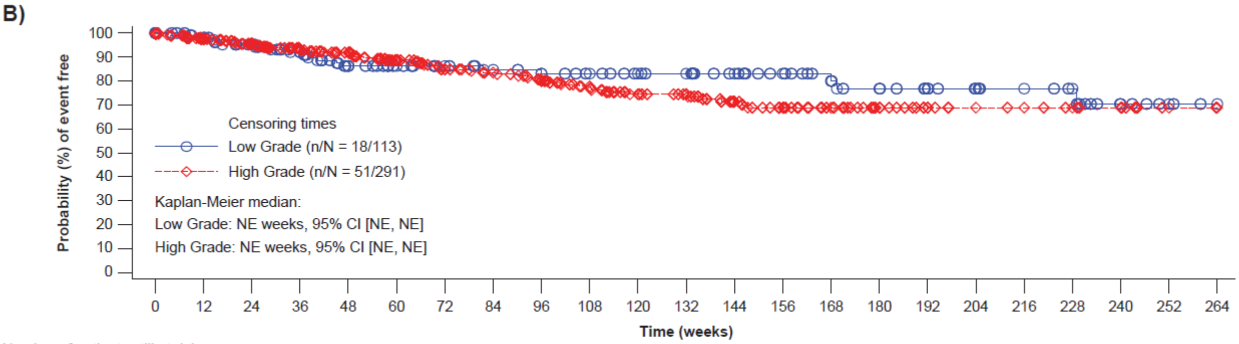

Number of patients still at risk	0	12	24	36	48	60	72	84	96	108	120	132	144	156	168	180	192	204	216	228	240	252	264
Low Grade	113	103	94	84	71	63	58	54	53	49	45	43	37	32	27	23	19	16	14	12	5	2	0
High Grade	291	257	230	199	181	152	131	123	114	99	83	77	59	49	38	24	16	12	11	9	7	1	0

CI, confidence interval; NE, not estimable; PMF, primary myelofibrosis.

Supplementary Figure S5. Overall survival in patients with PMF stratified by time since last biopsy. **A**, Patients who started ruxolitinib treatment ≤2 years since their last biopsy. **B**, Patients who started ruxolitinib treatment >2 years since their last biopsy.

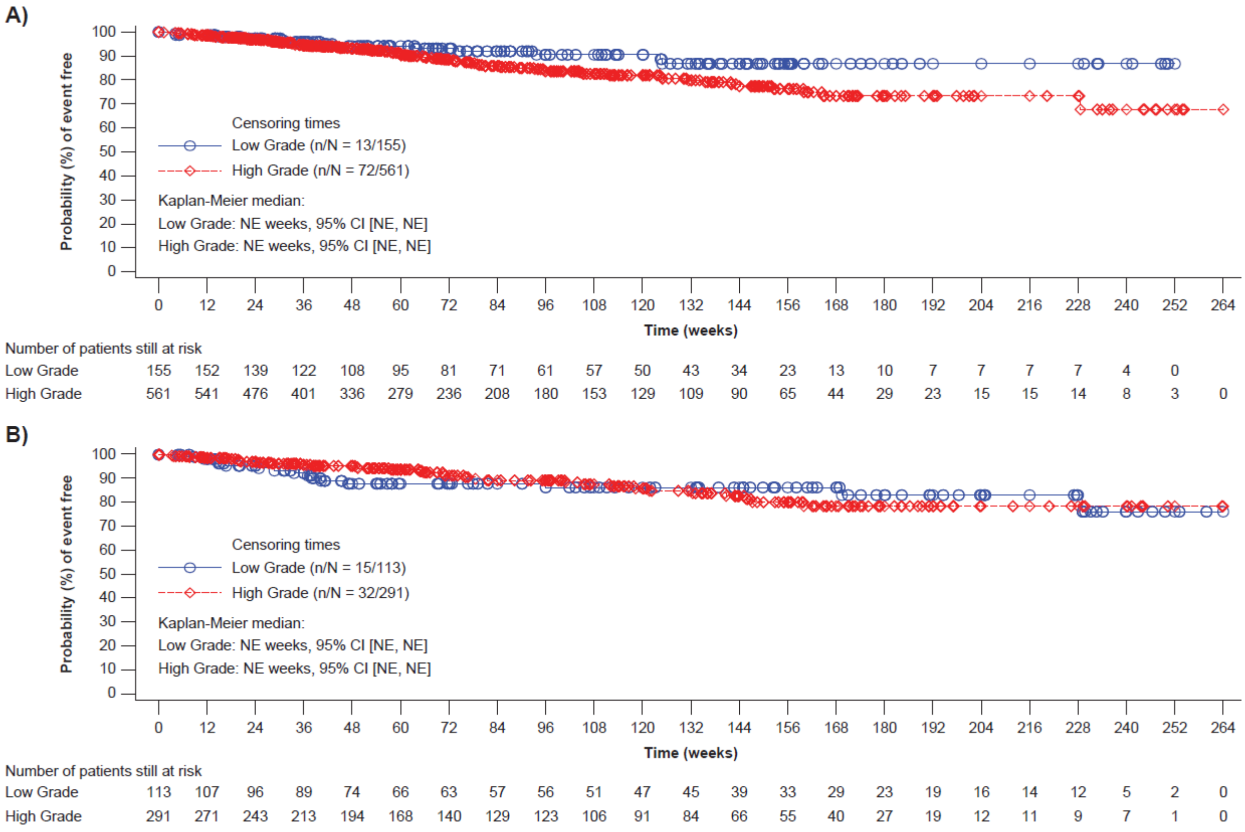

CI, confidence interval; NE, not estimable; PMF, primary myelofibrosis.
