# Supplementary material for: Benefit of Early Ruxolitinib Initiation Regardless of Fibrosis Grade in Patients with Primary Myelofibrosis: A Post Hoc Analysis of the Single-Arm Phase 3b JUMP Study
Source: Cancers (Basel). 2023 May 22;15(10):2859. doi: 10.3390/cancers15102859 (PMC10216208; doi:10.3390/cancers15102859)
Supplement: Supplementary file 1 [file cancers-15-02859-s001.zip › File S1. List of IRB-IECs_JUMP manuscript.pdf]

Study reference for approval: INC424A2401

**Center No.**	**Ethics Committee or Institutional Review Board**	**Department / Organization**	**City, State/Province, Postal Code Country**
1	Ottawa Hospital Research Ethics Board	725 Parkdale Avenue, Loeb building	Ottawa ON K1Y 1J7 Canada
2	UBC Providence Health Care Research Institute	1081 Burrard Street, 11th Floor, Hornby Site	Vancouver BC V6Y 1Y6 Canada
4	Ontario Cancer Research Ethics Board	101 College Street, MaRs Centre, South Tower, Room 800	Toronto ON M5G 0A3 Canada
5	Comite d' Ethique de la recherche du CHU de Quebec	10 Rue de L'Espinay, Local AO-124	Quebec QC G1L 3L5 Canada
6	McGill Faculty of Medicine Institutional Review Board	3655 Promenade Sir William Osler	Montreal QC H3G 1Y6 Canada
7	Capital health Research Ethics Bord	5790 University ave.	Halifax NS B3H 1V7 Canada
8	Ontario Cancer Research Ethics Board	101 College Street, MaRs Centre, South Tower, Room 800	Toronto ON M5G 0A3 Canada
9	Ontario Cancer Research Ethics Board	101 College Street, MaRs Centre, South Tower, Room 800	Toronto ON M5G 0A3 Canada
10	Biomedical Research Ethics Board (Bio-REB) University of Saskatchewan	1607 - 110 Gymnasium Place	Saskatoon SK S7N 4J8 Canada
11	Horizon Health Network Research Ethics Board	400 University Avenue	Moncton NB E1C 6Z8 Canada
12	HREBA Cancer Committee	10104 103 Avenue, Room 1500	Edmonton AB T5J 4A7 Canada
50	Siriraj Institutional Review Board	Faculty of Medicine, Siriraj Hospital	2, Wanglang Rd., Kwaeng Siriraj, Khet Bangkok Noi Bangkok Thailand
51	Med Chula IRB	Reserch Affairs, Faculty of Medicine, Chulalongkorn Hospital	Pathumwan Bangkok Thailand
52	Research Affairs	Faculty of Medicine, Ramathibodi Hospital	Research and Welfare Buiding, 3rd fl, Ratchathewi Bangkok Thailand
53	Research Affairs	Faculty of Medicine, Chiang Mai University	110, Inthawaroros Rd., Sriphum, Muang Chiang Mai Thailand
90	EC CHU Beni Messous	Ophtalmology	Algiers Algeria

120	Comite D'Ethique Hopital La Rabta	Regional Patients Protection Committee<br>Centre National de Pharmacovigilance (CNPV) of Tunis	Tunis-Bab Souika<br>Tunisia
121	Comite d'Ethique CHU Farhat Hached	Regional Patients Protection Committee<br>Centre National de Pharmacovigilance (CNPV) of Tunis	Tunis-Bab Souika<br>Tunisia
122	Comite d'Ethique<br>HOPITAL<br>HEDI CHAKER	Regional Patients Protection Committee<br>Centre National de Pharmacovigilance (CNPV) of Tunis	Tunis-Bab Souika<br>Tunisia
123	Comite d'Ethique Hopital Militaire	Regional Patients Protection Committee<br>Centre National de Pharmacovigilance (CNPV) of Tunis	Tunis-Bab Souika<br>Tunisia
200	Ethic committee of Hadassah MC	NA	Jerusalem 91120<br>Israel
201	Helsinki committee, Rabin<br>MC	NA	Petach Tikva 49100<br>Israel
202	Ethic committee of Sourasky MC	NA	Tel Aviv 64239<br>Israel
203	Ethic committee of Bnai Zion MC	NA	Haifa 31048<br>Israel
204	Ethic committee of HaEmek<br>MC	NA	Afula 18101<br>Israel
205	Ethic committee of Sheba<br>MC	NA	Ramat Gan 52621<br>Israel
400	Federal Almazov North-West scientific research center ethic committee	NA	Saint-Petersburg 197341<br>Russian Federation
401	Russian Research institute<br>of transfusiology and Hematology Ethic committee	NA	Saint-Petersburg 191024<br>Russian Federation
402	Novosibirsk medical university Ethic Committee	NA	Novosibirsk 630051<br>Russian Federation
404	Irkutsk Regional Hospital Ethic committee	NA	Irkutsk 664528<br>Russian Federation
405	Nizhniy Novgorod Regional<br>hospital I.M. Semashko Ethic committee	NA	Nizhniy Novgorod 603126<br>Russian Federation
406	Hematology scientific center Ethic Committee	NA	Moscow125167<br>Russian Federation

407	Rostov Medical University<br>Ethic committee	NA	Rostov-on-Don344022<br>Russian Federation
600	The South African Medical<br>Association (SAMAREC)	N/A	Pretoria Gauteng<br>South Africa
601	The South African Medical<br>Association (SAMAREC)	N/A	Pretoria Gauteng<br>South Africa
603	SAMAREC	N/A	Pretoria Gauteng<br>South Africa
604	Human Research Ethics Committee (HREC), University of Witwatersrand	Medical	Johannesburg Gauteng<br>South Africa
605	SAMAREC	N/A	Pretoria Gauteng<br>South Africa
900	Ethikkommission der Medizinischen Universitat<br>Wien	Borschkegasse 8b/E06	Vienna<br>Austria
901	Ethikkommission des Landes Salzburg	Michael-Pacher-Strasse 36 (EG, Zi.48)	Salzburg<br>Austria
902	Ethikkommission der Medizinischen Universitat<br>Innsbruck	Innrain 43	Innsbruck<br>Austria
903	Ethikkommission der Medizinischen Universitat<br>Wien	Borschkegasse 8b/E06	Vienna<br>Austria
904	Ethikkommission der Med.-<br>Universitat Graz	Auenbruggerplatz 2, 3.OG	Graz<br>Austria
905	Ethikkommission des Krankenhaus der Barmherzigen Schwestern<br>Linz	Seilerstatte 4	Linz<br>Austria
906	Ethikkommission des Krankenhaus der Elisabethinen Linz GmbH	Fadingerstrasse 1	Linz<br>Austria
1017	Eticka komise Fakultni nemocnice Olomouc	NA	Olomouc 775 20<br>Czech Republic
1018	Eticka komise Fakultni nemocnice Brno	NA	Brno 625 00<br>Czech Republic
1019	Eticka komise UHKT	NA	Praha 128 20<br>Czech Republic
1020	Eticka komise Fakultni nemocnice Hradec Kralova	NA	Hradec Kralova 500 05<br>Czech Republic
1100	Ethikkommission der Arztekammer Westfalen-Lippe und der Medizinischen	NA	Munster NA<br>Germany

	Fakultat der Westfalischen Wilhelms-Universitat Munster		
1101	Ethikkommission an der Medizinischen Fakultat der RWTH Aachen	NA	Aachen NA Germany
1102	Universitatsklinikum Jena Ethik-Kommission	NA	Jena NA 07740 Germany
1103	Ethik-Kommission der Medizinischen Fakultat Heidelberg	NA	Heidelberg NA Germany
1104	Technische Universitat Munchen Fakultat fur Medizin Ethikkommission	NA	Munchen NA Germany
1105	Otto-von-Guericke-Universitat Magdeburg Ethikkommission an der Medizinischen Fakultat UniversitAtsklinikum	NA	Magdeburg NA 39120 Germany
1106	Sachsische LandesArztekammer Ethikkommission	NA	Dresden NA 01009 Germany
1107	Arztekammer Hamburg Ethikkommission	NA	Hamburg NA Germany
1108	Landesamt fur Gesundheit und Soziales Ethikkommission des Landes Berlin	NA	Berlin NA Germany
1109	Albert-Ludwigs-Universitat Freiburg Ethikkommission	NA	Freiburg NA Germany
1110	Ethik-Kommission des Fachbereichs Medizin der Johann Wolfgang Goethe-Universitat Universitatsklinikum	NA	Frankfurt NA Germany
1111	Arztekammer Nordrhein Ethikkommission	NA	Dusseldorf NA Germany
1112	Ethikkommission der Arztekammer Westfalen-Lippe und der Medizinischen Fakultat der Westfalischen Wilhelms-Universitat Munster	NA	Munster NA Germany
1113	Ethikkommission des Landes Bremen Klinikum Bremen-Mitte gGmbH	NA	Bremen NA Germany

	Institut fur Pharmakologie		
1114	Ethik-Kommission der Universitatsklinikum Essen<br>Medizinische Fakultat der<br>Universitat Duisburg-Essen	NA	Essen NA<br>Germany
1115	Landesamt fur Gesundheit<br>und Soziales<br>Ethikkommission des Landes Berlin	NA	Berlin NA<br>Germany
1116	Ethikkommission an der Medizinischen Fakultat der<br>Universitat Leipzig	NA	Leipzig NA 04107<br>Germany
1117	LandesArztekammer Rheinland-Pfalz<br>Ethikkommission	NA	Mainz NA<br>Germany
1118	Ethikkommission bei der LandesArztekammer<br>Baden-Wurttemberg	NA	Stuttgart NA<br>Germany
1119	Geschaftsstelle Ethikkommission<br>Universitat zu Koln	NA	Koln NA<br>Germany
1120	Universitat Rostock<br>Medizinische Fakultat<br>Ethikkommission	NA	Rostock NA<br>Germany
1121	Ludwig-Maximilians-Universitat Munchen<br>Klinikum der Universitat<br>Ethik-Kommission	NA	Munchen NA<br>Germany
1122	Ethik-Kommission der Medizinischen Fakultat der<br>Christian-Albrechts-Universitat zu Kiel	NA	Kiel NA<br>Germany
1123	Universitat Ulm<br>Ethik-Kommission	NA	Ulm NA<br>Germany
1124	Ethik-Kommission der Medizinischen Fakultat der<br>Heinrich-Heine-Universitat<br>Dusseldorf	NA	Dusseldorf NA<br>Germany
1125	Arztekammer Nordrhein<br>Ethikkommission	NA	Dusseldorf NA<br>Germany
1127	Ethik-Kommission der Bayerischen<br>Landesarztekammer	NA	Munchen NA<br>Germany
1128	Arztekammer Nordrhein<br>Ethikkommission	NA	Dusseldorf NA<br>Germany

1129	Ethikkommission der Arztekammer Westfalen-Lippe und der Medizinischen Fakultat der Westfalischen Wilhelms-UniversitAt Munster	NA	Munster NA Germany
1130	Universitatsmedizin Greifswald Ethikkommission Institut fur Pharmakologie	NA	Greifswald NA Germany
1131	Ethik-Kommission der Arztekammer Niedersachsen	NA	Hannover NA Germany
1132	Ethikkommission der Arztekammer Westfalen-Lippe und der Medizinischen Fakultat der Westfalischen Wilhelms-UniversitAt Munster	NA	Munster NA Germany
1133	Arztekammer Nordrhein Ethikkommission	NA	Dusseldorf NA Germany
1135	Ethik-Kommission der Bayerischen LandesArztekammer	NA	Munchen NA Germany
1136	LandesArztekammer Hessen Ethik-Kommission	NA	Frankfurt NA Germany
1137	Landesamt fur Verbraucherschutz Ethik-Kommission des Landes Sachsen-Anhalt	NA	Dessau-Rosslau NA Germany
1138	Ethikkommission der Arztekammer Westfalen-Lippe und der Medizinischen Fakultat der Westfalischen Wilhelms-Universitat Munster	NA	Munster NA Germany
1139	Ethik-Kommission der LandesArztekammer Brandenburg	NA	Cottbus NA 03044 Germany
1141	Ethikkommission bei der LandesArztekammer Baden-Wurttemberg	NA	Stuttgart NA Germany
1142	Ethik-Kommission der UniversitAt Gottingen Medizinische Fakultat	NA	Gottingen NA Germany
1143	Ethik-Kommission der Medizinischen	NA	Hannover NA Germany

	Hochschule Hannover		
1144	LandesArztekammer Hessen Ethik-Kommission	NA	Frankfurt NA Germany
1146	Ethik-Kommission der Arztekammer Niedersachsen	NA	Hannover NA Germany
1147	Ethik-Kommission der Bayerischen LandesArztekammer	NA	Munchen NA Germany
1149	Arztekammer Hamburg Ethikkommission	NA	Hamburg NA Germany
1150	Ethik-Kommission der Bayerischen LandesArztekammer	NA	Munchen NA Germany
1152	LandesArztekammer Hessen Ethik-Kommission	NA	Frankfurt NA Germany
1153	Philipps-Universitat Marburg Fachbereich Medizin Dekanat/Ethikkommissio n	NA	Marburg NA Germany
1154	Sachsische LandesArztekammer Ethikkommission	NA	Dresden NA 01009 Germany
1156	Ethik-Kommission der Bayerischen LandesArztekammer	NA	Munchen NA Germany
1159	Friedrich-Alexander-Universitat Erlangen-Nurnberg Medizinische Fakultat Ethik-Kommission	NA	Erlangen NA Germany
1162	Ethik-Kommission der Bayerischen LandesArztekammer	NA	Munchen NA Germany
1163	Arztekammer Nordrhein Ethikkommission	NA	Dusseldorf NA Germany
1166	Ethik-Kommission der Arztekammer Niedersachsen	NA	Hannover NA Germany
1169	Ethik-Kommission der Arztekammer Niedersachsen	NA	Hannover NA Germany
1300	Regionalis es Intezeti Kutatasetikai Bizottsag	NA	Szombathely H-9700 Hungary
1301	Regionalis Human Orvosbiologiai Kutatasetikai Bizottsag	NA	Szeged H-6725 Hungary
1303	Regionalis es Intezmenyi Kutatasetikai Bizottsag	NA	Debrecen H-4032 Hungary

1304	Kutatasetikai Bizottsag	NA	Budapest H-1097<br>Hungary
1306	Intezeti Kutatasetikai Bizottsag	NA	Kaposvar H-7400<br>Hungary
1400	COMITATO ETICO AREA<br>VASTA CENTRO	AZIENDA OSPEDALIERO UNIVERSITARIA<br>CAREGGI DI FIRENZE	LARGO GIOVANNI ALESSANDRO BRAMBILLA, 3<br>Firenze (FI)<br>Italy
1401	COMITATO INDIPENDENTE DI ETICA<br>MEDICA	AUSL BR/1 DI BRINDISI	VIA NAPOLI, 8 BRINDISI (BR)<br>Italy
1402	COMITATO ETICO DELL'AZIENDA OSPEDALIERO-UNIVERSITARIA POLICLINICO - VITTORIO EMANUELE DI CATANIA -<br>per POU Vittorio Emanuele, Ferrarotto, Santo Bambino	COMITATO ETICO DELL'AZIENDA OSPEDALIERO-UNIVERSITARIA POLICLINICO - VITTORIO EMANUELE DI CATANIA - per POU Vittorio<br>Emanuele, Ferrarotto, Santo Bambino	VIA GESUALDO CLEMENTI, 36<br>CATANIA (CT)<br>Italy
1403	COMITATO ETICO PER LA<br>SPERIMENTAZIONE CLINICA DEI MEDICINALI<br>AREA VASTA SUD EST	AZIENDA OSPEDALIERA UNIVERSITARIA<br>SENESE DI SIENA	c/o UOC Farmacia AOUS - Viale<br>Bracci SIENA (SI)<br>Italy
1404	COMITATO ETICO DELLA<br>PROVINCIA DI MODENA	C/O AZIENDA POLICLINICO	VIA DEL POZZO 71<br>MODENA (MO)<br>Italy
1405	COMITATO ETICO PROVINCIALE DI REGGIO<br>EMILIA	c/o Azienda<br>Ospedaliera Arcispedale S. Maria Nuova/IRCCS - I-RS<br>Edificio Spallanzani	Viale Umberto I, 50<br>REGGIO<br>EMILIA (RE)<br>Italy
1406	COMITATO ETICO DELL`AZIENDA OSPEDALIERA POLICLINICO<br>CONSORZIALE DI BARI	COMITATO ETICO DELL`AZIENDA OSPEDALIERA POLICLINICO<br>CONSORZIALE DI BARI	PIAZZALE GIULIO CESARE, 11<br>BARI (BA)<br>Italy
1407	COMITATO ETICO DELL'AZIENDA OSPEDALIERA BIANCHI - MELACRINO - MORELLI DI REGGIO CALABRIA	COMITATO ETICO DELL'AZIENDA OSPEDALIERA BIANCHI - MELACRINO - MORELLI DI REGGIO CALABRIA	VIA SPIRITO SANTO 24 REGGIO<br>DI CALABRIA (RC)<br>Italy
1408	COMITATO ETICO DELLE<br>AZIENDE SANITARIE DELL'UMBRIA	SEGRETERIA SCIENTIFICO-AMMINISTRATIVA DEL CEAS UMBRIA	Via della Rivoluzione, 16 - ELLERA DI CORCIANO<br>CORCIANO (PG)<br>Italy

1409	COMITATO ETICO DELL'AZIENDA OSPEDALIERA A.CARDARELLI NAPOLI	COMITATO ETICO DELL'AZIENDA OSPEDALIERA A.CARDARELLI NAPOLI	Via Cardarelli Antonio, 9 NAPOLI (NA) Italy
1410	COMITATO ETICO INDIPENDENTE	FONDAZIONE PTV POLICLINICO TOR VERGATA DI ROMA	VIALE OXFORD, 81 ROMA (RM) Italy
1411	COMITATO ETICO PROVINCIALE DI VARESE	C/O OSPEDALE DI CIRCOLO E FONDAZIONE MACCHI	VIALE LUIGI BORRI 57 VARESE (VA) Italy
1412	COMITATO ETICO REGIONALE UNICO (CERU)	c/o AZIENDA OSPEDALIERO-UNIVERSITARIA S. MARIA DELLA MISERICORDIA DI UDINE	Piazzale S. MARIA DELLA MISERICORDIA, 15 UDINE (UD) Italy
1413	COMITATO ETICO PER LA SPERIMENTAZIONE CLINICA DELLE PROVINCE DI VERONA E ROVIGO	AOUI VERONA	PIAZZALE LUDOVICO ANTONIO SCURO, 10 VERONA (VR) Italy
1414	COMITATO ETICO AREA DI PAVIA	FONDAZIONE IRCCS POLICLINICO SAN MATTEO DI PAVIA	VIALE GOLGI 19 PAVIA (PV) Italy
1415	COMITATO ETICO MILANO AREA C	c/o Azienda Ospedaliera Ospedale Niguarda Ca' Granda - Padiglione 7, Ala D, 4Â° piano	PIAZZA DELL' OSPEDALE MAGGIORE, 3 MILANO (MI) Italy
1416	COMITATO ETICO DELL'UNIVERSITA' SAPIENZA	COMITATO ETICO DELL'UNIVERSITA' SAPIENZA	VIALE DEL POLICLINICO, 155 ROMA (RM) Italy
1417	COMITATO ETICO DEGLI ISTITUTI FISIOTERAPICI OSPITALIERI (IRCCS) DI ROMA	COMITATO ETICO DEGLI ISTITUTI FISIOTERAPICI OSPITALIERI (IRCCS) DI ROMA	VIA ELIO CHIANESI 53 ROMA (RM) Italy
1418	COMITATO ETICO LAZIO 1	ZIENDA OSPEDALIERA SAN CAMILLO - C. FORLANINI DI ROMA	CIRCONVALLAZIONE GIANICOLENSE, 87 ROMA (RM) Italy
1419	COMITATO ETICO REGIONE LIGURIA	c/o IRCCS AOU SAN MARTINO - IST ISTITUTO NAZIONALE PER LA RICERCA SUL CANCRO	LARGO ROSANNA BENZI, 10 GENOVA (GE) Italy
1420	COMITATO ETICO REGIONE LIGURIA	c/o IRCCS AOU SAN MARTINO - IST ISTITUTO NAZIONALE PER LA RICERCA SUL CANCRO	LARGO ROSANNA BENZI, 10 GENOVA (GE) Italy
1421	COMITATO ETICO INDIPENDENTE	AZIENDA OSPEDALIEROUNIVERSITARI	Via Albertoni Pietro, 15 - Padiglione

		A<br>POLICLINICO S.ORSOLA -<br>MALPIGHI DI BOLOGNA	3 BOLOGNA (BO)<br>Italy
1422	COMITATO ETICO<br>DELLE<br>PROVINCE DI TREVISO<br>E<br>BELLUNO	AZIENDA ULSS 9 TREVISO	VIA SANT'AMBROGIO DI<br>FIERA,<br>37 TREVISO (TV)<br>italy
1423	COMITATO ETICO PER<br>LE<br>SPERIMENTAZIONI<br>CLINICHE DELLA<br>PROVINCIA DI<br>VENEZIA E<br>IRCCS SAN CAMILLO	ULSS 12<br>VENEZIANA	VIA DON FEDERICO<br>TOSATTO,<br>147 VENEZIA (VE)<br>Italy
1424	COMITATO ETICO<br>AREA<br>DI PAVIA -<br>FONDAZIONE<br>IRCCS POLICLINICO<br>SAN MATTEO DI PAVIA	COMITATO ETICO AREA DI<br>PAVIA - FONDAZIONE IRCCS<br>POLICLINICO<br>SAN MATTEO DI PAVIA	VIALE GOLGI 19 PAVIA<br>(PV)<br>Italy
1425	COMITATO ETICO<br>DELL'AZIENDA<br>OSPEDALIERA<br>UNIVERSITARIA<br>MAGGIORE DELLA<br>CARITA' DI NOVARA	COMITATO ETICO<br>DELL'AZIENDA OSPEDALIERA<br>UNIVERSITARIA<br>MAGGIORE DELLA CARITA' DI<br>NOVARA	Corso Mazzini 18 NOVARA<br>(NO)<br>Italy
1426	COMITATO ETICO<br>DELL'OSPEDALE<br>ONCOLOGICO<br>REGIONALE DI<br>RIONERO<br>IN VULTURE (PZ)	COMITATO ETICO<br>DELL'OSPEDALE<br>ONCOLOGICO<br>REGIONALE DI RIONERO IN<br>VULTURE (PZ)	VIA PADRE PIO, 1<br>RIONERO IN<br>VULTURE (PZ)<br>Italy
1427	COMITATO ETICO<br>DELLA<br>AUSL LE DI LECCE	COMITATO ETICO DELLA<br>AUSL LE DI LECCE	Via Miglietta 5 LECCE (LE)<br>Italy
1428	COMITATO di Bioetica<br>Palermo 1	c/o AZIENDA OSPEDALIERA<br>UNIVERSITARIA POLICLINICO<br>P. GIACCONE	VIA DEL VESPRO, 127<br>PALERMO<br>(PA)<br>Italy
1429	COMITATO ETICO PER<br>LE<br>ATTIVITA' BIOMEDICHE<br>CARLO ROMANO<br>DELL'UNIVERSITA'<br>DEGLI<br>STUDI FEDERICO II DI<br>NAPOLI	COMITATO ETICO PER LE<br>ATTIVITA' BIOMEDICHE<br>CARLO ROMANO<br>DELL'UNIVERSITA' DEGLI<br>STUDI FEDERICO II DI NAPOLI	VIA SERGIO PANSINI, 5<br>NAPOLI<br>(NA)<br>Italy
1430	COMITATO ETICO ASL<br>NA/1 DI NAPOLI	COMITATO ETICO ASL NA/1 DI<br>NAPOLI	STRADA COMUNALE DEL<br>PRINCIPE, 13 NAPOLI (NA)<br>Italy
1431	COMITATO ETICO<br>UNICO<br>DELLA PROVINCIA DI<br>FERRARA	COMITATO ETICO UNICO<br>DELLA PROVINCIA DI<br>FERRARA	VIA ALDO MORO, 8<br>FERRARA<br>(FE)<br>italy

1432	COMITATO ETICO PER LE<br>PROVINCE DI CHIETI E PESCARA	COMITATO ETICO PER LE PROVINCE DI CHIETI E PESCARA	VIA DEI VESTINI, 31 CHIETI<br>SCALO (CH)<br>Italy
1433	COMITATO ETICO DELL'UNIVERSITA' CATTOLICA DEL S. CUORE	POLICLINICO GEMELLI, ROMA - LAZIO	LARGO GEMELLI 8 ROMA (RM)<br>Italy
1434	COMITATO ETICO LAZIO<br>2	ASL ROMA C	VIA PRIMO CARNERA, 1 ROMA<br>(RM)<br>Italy
1435	COMITATO ETICO PER LE<br>SPERIMENTAZIONI CLINICHE DELLA PROVINCIA DI PADOVA	AOU PADOVA	VIA NICOLO' GIUSTINIANI, 2<br>PADOVA (PD)<br>Italy
1436	COMITATO ETICO DELLE<br>AZIENDE SANITARIE DELL'UMBRIA	SEGRETERIA SCIENTIFICO-AMMINISTRATIVA<br>DEL CEAS UMBRIA	Via della Rivoluzione, 16 - ELLERA DI CORCIANO<br>CORCIANO (PG)<br>Italy
1437	COMITATO DI ETICA DELLA ASL DI SALERNO	COMITATO DI ETICA DELLA ASL DI SALERNO	VIA FEDERICO RICCO 50<br>NOCERA INFERIORE (SA)<br>Italy
1438	COMITATO ETICO DELLA<br>PROVINCIA DI BERGAMO	COMITATO ETICO DELLA PROVINCIA DI BERGAMO	PIAZZA OMS, 1 BERGAMO (BG)<br>Italy
1439	COMITATO ETICO INDIPENDENTE	AZIENDA OSPEDALIERA UNIVERSITARIA S. LUIGI GONZAGA<br>DI ORBASSANO	Via Regione Gonzole, 10<br>ORBASSANO (TO)<br>Italy
1440	COMITATO ETICO DELL'IRCCS ISTITUTO TUMORI GIOVANNI PAOLO II DI BARI	SEZIONE PRESSO LA FONDAZIONE CASA SOLLIEVO<br>DELLA<br>SOFFERENZA DI SAN GIOVANNI ROTONDO	VIALE CAPPUCCINI SAN GIOVANNI ROTONDO (FG)<br>italy
1441	COMITATO ETICO AZIENDA OSPEDALIERA UNIVERSITARIA DI CAGLIARI	COMITATO ETICO AZIENDA OSPEDALIERA UNIVERSITARIA DI CAGLIARI	VIA OSPEDALE, 54<br>CAGLIARI (CA)<br>Italy
1442	COMITATO ETICO AZIENDA OSPEDALIERA UNIVERSITARIA DI CAGLIARI	COMITATO ETICO AZIENDA OSPEDALIERA UNIVERSITARIA DI CAGLIARI	VIA OSPEDALE, 54<br>CAGLIARI (CA)<br>Italy
1443	COMITATO ETICO INTERAZIENDALE	AO SS. ANTONIO E BIAGIO E CESARE ARRIGO DI ALESSANDRIA	Via Venezia 16<br>ALESSANDRIA (AL)<br>Italy
1445	COMITATO ETICO PER LA<br>SPERIMENTAZIONE CLINICA DELLA	ULSS 6 VICENZA	VIALE F. RODOLFI, 37 VICENZA<br>(VI)<br>Italy

	PROVINCIA DI VICENZA		
1446	COMITATO ETICO PALERMO 2	AZIENDA OSPEDALIERA OSPEDALI RIUNITI VILLA SOFIA - CERVELLO	VIA TRABUCCO, 180 PALERMO (PA) Italy
1447	COMITATO ETICO LAZIO 2	ASL ROMA C	VIA PRIMO CARNERA, 1 ROMA (RM) Italy
1448	COMITATO ETICO IRST IRCCS AVR	COMITATO ETICO IRST IRCCS AVR	Via Piero Maroncelli, 40 MELDOLA (FC) Italy
1449	COMITATO ETICO DELLA FONDAZIONE IRCCS ISTITUTO NAZIONALE DEI TUMORI DI MILANO	COMITATO ETICO DELLA FONDAZIONE IRCCS ISTITUTO NAZIONALE DEI TUMORI DI MILANO	VIA GIACOMO VENEZIAN, 1 MILANO (MI) Italy
1450	COMITATO ETICO DELL'AZIENDA OSPEDALIERO-UNIVERSITARIA OSPEDALI RIUNITI UMBERTO I -GM LANCISI-G. SALESI DI ANCONA	COMITATO ETICO DELL'AZIENDA OSPEDALIERO-UNIVERSITARIA OSPEDALI RIUNITI UMBERTO I -GM LANCISI-G. SALESI DI ANCONA	Via Conca, Torrette ANCONA (AN) italy
1451	COMITATO ETICO DELL'AZIENDA OSPEDALIERA OSPEDALI RIUNITI MARCHE NORD	COMITATO ETICO DELL'AZIENDA OSPEDALIERA OSPEDALI RIUNITI MARCHE NORD	Piazzale Cinelli, 4 PESARO (PU) Italy
1452	COMITATO ETICO DELL'IRCCS ISTITUTO PER LO STUDIO E LA CURA DEI TUMORI	FONDAZIONE GIOVANNI PASCALE DI NAPOLI	Via Semmola Mariano NAPOLI (NA) Italy
1453	COMITATO ETICO DELL'AZIENDA OSPEDALIERA DI RILIEVO NAZIONALE E DI ALTA SPECIALIZZAZIONE GARIBALDI DI CATANIA	COMITATO ETICO DELL'AZIENDA OSPEDALIERA DI RILIEVO NAZIONALE E DI ALTA SPECIALIZZAZIONE GARIBALDI DI CATANIA	PIAZZA SANTA MARIA GESU', 5 CATANIA (CT) Italy
1454	COMITATO PER LA SPERIMENTAZIONE CLINICA DEI MEDICINALI AREA VASTA NORD OVEST	AZIENDA OSPEDALIERO UNIVERSITARIA PISANA DI PISA	VIA ROMA, 67 PISA (PI) Italy
1455	COMITATO ETICO MILANO AREA B	c/o FONDAZIONE IRCCS CA' GRANDA OSPEDALE MAGGIORE	VIA FRANCESCO SFORZA, 28

		POLICLINICO - PALAZZO UFFICI - 2Â° piano - c.a. dr.ssa Patti	MILANO (MI)<br>Italy
1456	COMITATO ETICO DELLA<br>PROVINCIA DI BRESCIA	COMITATO ETICO DELLA PROVINCIA DI BRESCIA	PIAZZALE SPEDALI CIVILI, 1<br>BRESCIA (BS)<br>Italy
1457	COMITATO ETICO DELLA<br>PROVINCIA DI MONZA BRIANZA	COMITATO ETICO DELLA PROVINCIA DI MONZA BRIANZA	VIA PERGOLESI 33<br>MONZA (MB)<br>Italy
1458	COMITATO ETICO DELL'AZIENDA OSPEDALIERA 'S. GIUSEPPE MOSCATI'<br>DI AVELLINO	COMITATO ETICO DELL'AZIENDA OSPEDALIERA 'S. GIUSEPPE MOSCATI'<br>DI AVELLINO	CONTRADA AMORETTA-CITTA'<br>OSPEDALIERA AVELLINO (AV)<br>Italy
1460	COMITATO ETICO INTERAZIENDALE A.O. CITTA' DELLA SALUTE E<br>DELLA SCIENZA DI TORINO - AO MAURIZIANO DI TORINO	ASL TO1	CORSO BRAMANTE, 88/90<br>TORINO (TO)<br>Italy
1461	COMITATO ETICO LAZIO 1	AZIENDA OSPEDALIERA SAN CAMILLO<br>- C. FORLANINI DI ROMA	CIRCONVALLAZIONE GIANICOLENSE, 87 ROMA (RM)<br>Italy
1462	COMITATO ETICO DELL'AZIENDA OSPEDALIERA PUGLIESE<br>- CIACCIO DI CATANZARO	COMITATO ETICO DELL'AZIENDA OSPEDALIERA PUGLIESE - CIACCIO DI CATANZARO	VIA VINICIO CORTESE, 25<br>CATANZARO (CZ)<br>Italy
1600	Niezalezna Komisja Bioetyczna ds. Badan Naukowych	Gdanski Uniwersytet Medyczny	Gdansk Pomorskie 80-211<br>Poland
1602	Komisja Bioetyczna	Opolska Izba Lekarska	Opole Opolskie 45-054<br>Poland
1603	Komisja Bioetyczna	Slaski Uniwersytet Medyczny	Katowice Slaskie 40-055<br>Poland
1604	Komisja Bioetyczna	Uniwersytet Mikolaja Kopernika w Toruniu	Bydgoszcz Kujawsko-Pomorskie 85-<br>094<br>Poland
1605	Komisja Bioetyczna	Uniwersytet Jagiellonski	Krakow Malopolskie 31-531<br>Poland
1606	Komisja Bioetyczna	Instytut Hematologii i Transfuzjologii	Warszawa Mazowieckie 02-776<br>Poland
1700	Conselho de Administraçao	CHTMAD - Hospital Vila Real	Vila Real 5000-508<br>Portugal
1701	Conselho de Administraçao	CENTRO HOSPITALAR DO PORTO, EPE - HSA	Porto 4099-001<br>Portugal

1702	Conselho de Administraçao	Centro Hospitalar Lisboa Norte, E.P.E.	Lisboa 1649-035 Portugal
1703	Conselho de Administraçao	Centro Hospitalar do Algarve, E.P.E.	Faro 8000-386 Portugal
1704	Conselho de Administraçao	Centro Hospitalar e Universitario de Coimbra, E.P.E	Coimbra 3000-075 Portugal
1801	Eticka komisia Univerzitna nemocnica Bratislava Nemocnica sv. Cyrila a Metoda	NA	Bratislava Slovakia SK-85107 Slovakia
1900	Comite Etico de Investigacion Hospital Regional Universitario de Malaga	Unidad Garantia de Calidad	Malaga Malaga Spain
1901	Comite Etico de Investigacion Clinica, Complejo Hospitalario de Jaen	NA	Jaen Jaen Spain
1902	COMITE ETICO DE INVESTIGACION Hospital Universitario Virgen Macarena	Unidad de Investigacion	Sevilla Sevilla Spain
1903	Comite de Etica de Investigacion del Hospital Universitario Virgen del Rocio	Ensayos Clinicos	Sevilla Sevilla Spain
1904	Comite de Etica de Investigacion del Hospital Universitario Puerta del Mar	Docencia MIR	Cadiz Cadiz Spain
1906	Comite Etico de Investigacion Clinica de Aragon (CEICA)	Instituto Aragones de Ciencias de la Salud	Zaragoza Zaragoza Spain
1907	Comite Etico de Investigacion Clinica de Aragon (CEICA)	Instituto Aragones de Ciencias de la Salud	Zaragoza Zaragoza Spain
1908	Comite de Etica de la Investigacion del Principado de Asturias	NA	Oviedo Asturias Spain
1909	CEIC del Hospital General Universitario de Alicante	NA	Alicante Alicante 03010 Spain
1910	CEIC del Hospital Clinico Universitario de Valencia	Secretaria	Valencia Valencia Spain
1911	CEIC del Hospital Universitario La Fe	Secretaria del CEIC	Valencia Valencia Spain
1912	CEIC de Cantabria	Secretaria Tecnica	Santander Cantabria Spain
1913	Comite Etico de Investigacion Clinica del	NA	Barcelona Barcelona 08036 Spain

	Hospital Clinic de Barcelona		
1914	Comite Etico de Investigacion Clinica del Parc de Salut Mar	NA	Barcelona Barcelona 08003 Spain
1915	Comite Etico de Investigacion Clinica del Hospital Universitari Germans Trias i Pujol	Secretaia Tecnica	Badalona Barcelona 08916 Spain
1916	Comite de Etica de Investigacion Clinica Hospital Universitari de Bellvitge	Secretaria Administrativa	Hospitalet de Llobregat Barcelona 08907 Spain
1917	CEIC Hospital Universitari Vall d’Hebron	Unidad de Soporte al CEIC	Barcelona Barcelona 08035 Spain
1918	Comite Etico de Investigacion Clinica Hospital Universitari de Tarragona Joan XXIII	Secretaria Tecnica-Admisnitrativa del CEIC	Tarragona Tarragona Spain
1919	Comite d'Etica d'investigacio Clinica (CEIC) de l'Hospital Universitari Doctor Josep Trueta, de Girona	NA	Girona Girona Spain
1921	CEIC Complejo Hospitalario de Toledo	Antigua Escuela de Enfermeras	Toledo Toledo Spain
1923	Comite Autonomico de Etica de la Investigacion de Galicia - SERGAS	Subdireccion de Farmacia y Productos Sanitarios Conselleria de Sanidade	Santiago de Compostela La Coruña Spain
1925	COMITE ETICO DE INVESTIGACION CLINICA HOSPITAL UNIVERSITARIO DE SALAMANCA	NA	Salamanca Salamanca Spain
1926	CEIC Complejo Asistencial de Burgos	Unidad de Investigacion	Burgos Burgos 09006 Spain
1928	Comite de Etica de la Investigacion de las Illes Balears (CEI-IB)	Direccion General de Salud Publica y Consumo	Palma de Mallorca Islas Baleares 07010 Spain
1929	Comite Etico de Investigacion Clinica HOSPITAL DE GRAN CANARIA DOCTOR NEGRIN	Secretaria CEIC	Las Palmas de Gran Canaria Las Palmas Spain
1931	Comite Etico de Investigacion Clinica HOSPITAL UNIVERSITARIO LA PRINCESA	NA	Madrid Madrid Spain

1933	Instituto de Investigacion Hospital 12 de Octubre	Unidad de Gestion Economica-Facturacion/Ingresos	Madrid Madrid Spain
1934	CEIC Hospital Univ. Puerta de Hierro de Majadahonda	Secretaria Tecnica del CEIC	Majadahonda Madrid Spain
1935	CEIC Aea 3 Direccion Medica HOSPITAL UNIVERSITARIO PRINCIPE DE ASTURIAS DE ALCALA DE HENARES	Direccion Medica	Alcala de Henares Madrid Spain
1936	Comite Etico de Investigacion Clinica HOSPITAL UNIVERSITARIO LA PAZ	NA	Madrid Madrid Spain
1937	CEIC Area 7 - Hospital Clinico San Carlos de Madrid	Servicio de Farmacologia Clinica	Madrid Madrid Spain
1938	CEIC Hospital Univ. Puerta de Hierro de Majadahonda	Secretaria Tecnica del CEIC	Majadahonda Madrid Spain
1939	CEIC DEL HOSPITAL VIRGEN DE LA ARRIXACA	NA	El Palmar Murcia Spain
1940	Comite Etico de Investigacion Clinica de Navarra	Secretaria Tecnica del CEIC	Pamplona Navarra Spain
1941	Comite Etico de Investigacion Clinica de Euskadi (CEIC-E)	Secretaria del CEIC	Vitoria Alava 01010 Spain
1942	Comite Etico de Investigacion Clinica de Euskadi (CEIC-E)	Secretaria del CEIC	Vitoria Alava 01010 Spain
1943	Comite Etico de Investigacion Clinica HOSPITAL UNIVERSITARIO LA PAZ	NA	Madrid Madrid Spain
1944	Comite Etico de Investigacion Clinica HOSPITAL UNIVERSITARIO LA PAZ	NA	Madrid Madrid Spain
1946	Comite Etico de Investigacion Clinica HOSPITAL RAMON Y CAJAL	NA	Madrid Madrid Spain
1947	COMITE ETICO DE INVESTIGACION CLINICA HOSPITAL DE LA	NA	Barcelona Barcelona 08025 Spain

	SANTA<br>CREU I SANT PAU		
1948	COMITE ETICO DE INVESTIGACION CLINICA<br>HOSPITAL DE LA SANTA<br>CREU I SANT PAU	NA	Barcelona Barcelona 08025<br>Spain
1949	COMITE ETICO DE INVESTIGACION CLINICA<br>HOSPITAL DE LA SANTA<br>CREU I SANT PAU	NA	Barcelona Barcelona 08025<br>Spain
1950	Comite Etico de Investigacion Clinica de la Rioja	Consejeria de Salut	Logroño Logroño<br>Spain
1951	CEIC Corporacio Sanitaria Parc Taulet	NA	Sabadell Barcelona 08208<br>Spain
1952	CEIC del Hospital Universitario de Canarias	NA	La Laguna Tenerife<br>Spain
1953	Comite Autonomico de Etica de la Investigacion de Galicia - SERGAS	Subdireccion de Farmacia y Productos Sanitarios Conselleria de Sanidade	Santiago de Compostela La Coruña<br>Spain
1954	Comite Etico de Investigacion Clinica de Aragon (CEICA)	Instituto Aragones de Ciencias de la Salud	Zaragoza Zaragoza<br>Spain
1955	Comite Etico de Investigacion<br>Hospital Regional Universitario de Malaga	Secretaria del CEIC	Malaga Malaga<br>Spain
2102	GENERAL HOSPITAL OF ATHENS "EVAGELISMOS"	HEMATOLOGY CLINIC	ATHENS IPSILANTOU 45-47<br>GREECE
2104	GENERAL HOSPITAL OF ATHENS "LAIKO"	1ST PROP.INT.MEDICINE CLINIC	ATHENS AGIOU THOMA 17<br>GREECE
2107	GENERAL HOSPITAL OF ATHENS "GENIMATAS"	HEMATOLOGY DPT	ATHENS MESOGEION AV. 154<br>GREECE
2108	UNIVERSITY GENERAL HOSPITAL OF PATRAS	HEMATOLOGY DPT	PATRA RIO PATRAS<br>GREECE
3100	Institutional Review board, King Abdullah International<br>Medical Research Center	Research Center	Riyadh<br>Saudi Arabia
3101	Institutional Review board, King Abdullah International<br>Medical Research Center	Research Center	Riyadh<br>Saudi Arabia
3160	Comite d'Ethique pour la Recherche Biomedicale	Departement de pharmacologie	Casablanca Casablanca<br>Morocco
3530	Ethics and Medical and Research Department,	St Vincent's Healthcare Group Ltd	Dublin D4<br>Ireland

	Education & Research Centre,		
4000	Comite Independiente de Etica para Ensayos en Farmacologia Clinica	NA	CABA CABA C1027AAP<br>Argentina
4001	Comite Independiente de Etica para Ensayos en Farmacologia Clinica	NA	CABA CABA C1027AAP<br>Argentina
4002	Comite Independiente de Etica para Ensayos en Farmacologia Clinica	NA	CABA CABA C1027AAP<br>Argentina
4003	Comite de Etica en Investigacion	NA	Buenos Aires Buenos Aires 609<br>(1221).<br>Argentina
4004	Comite de Revision Interna.<br>Hospital Privado	NA	Cordoba Cordoba X5016KEH<br>Argentina
4006	Comite de Etica en Investigacion Hospital Español Sociedad de Beneficiencia.	NA	CABA CABA 1209<br>Argentina
5000	Associaçao de Combate ao Cancer	EC	Goiania GO 74605-070<br>Brazil
5001	Casa de Saude Santa Marcelina	EC	Sao Paulo SP 06270-070<br>Brazil
5002	Hospital de Clinica da UFPR	EC	Curitiba PR 80060-900<br>Brazil
5003	Universidade Federal de Minas Gerais	EC	Belo Horizonte MG 31270-901<br>Brazil
5004	Hospital das Clinicas da Faculdade de Medicina de Ribeirao Preto da Universidade de Sao Paulo	EC	Ribeirao Preto SP 14048-900<br>Brazil
5005	Faculdade de Ciencias Medicas da UNICAMP	EC	Campinas SP 13083-970<br>Brazil
5006	Irmandade Santa Casa de Sao Paulo	EC	Sao Paulo SP 01221-010<br>Brazil
5007	Hospital de Clinicas de Porto Alegre - HCPA/UFRGS	EC	Porto Alegre RS 90035-903<br>Brazil
5008	Hospital Israelita Albert Einstein	EC	Sao Paulo SP 05652-000<br>Brazil
5009	Hemorio - RJ	EC	Rio de Janeiro RJ 20211-090<br>Brazil
6010	CREIMED Comite de Estudios Medicos SAS	Antioquia	Medellin Antioquia<br>Colombia
6011	Comite de Etica en Investigacion con Seres Humanos	Bogota D.C.	Bogota D.C. Bogota D.C.<br>Colombia
7011	Comite de Etica en Investigacion del Instituto	NA	Ciudad de Mexico Ciudad de

	Mexicano del Seguro Social, Coordinacion de Investigacion en Salud		Mexico<br>Mexico
7012	Comite de Etica en Investigacion del Hospital General de Mexico Dr. Eduardo Liceaga	NA	Ciudad de Mexico Ciudad de<br>Mexico<br>Mexico
7013	Comite de Etica en Investigacion del Hospital Dr. Jose Eleuterio Gonzalez<br>de la Facultad de Medicina y Hospital Universitario de<br>la Universidad Autonoma de Nuevo Leon	NA	Monterrey Nuevo Leon<br>Mexico
7014	Comite de Etica en Investigacion del Hospital General del Estado de Sonora Dr. Ernesto Ramos Boursa	NA	Hermosillo Sonora<br>Mexico
7015	Comite de Etica en Investigacon del Hospital Civil de Guadalajara Fray Antonio Alcalde	NA	Guadalajara Jalisco<br>Mexico
9010	Commissie Medische Ethiek	Commissie Medische Ethiek	Leuven Vlaams Brabant<br>Belgium
9011	Ethisch Comite UZA	Ethisch Comite UZA	Edegem Antwerpen<br>Belgium
9013	Commissie voor Medische<br>Ethiek ZNA	Commissie voor medische ethiek ZNA	Antwerpen Antwerpen<br>Belgium
9014	Commissie Medische Ethiek	Commissie Medische Ethiek	Roeselare West Vlaanderen<br>Belgium
9015	Commissie Medische Ethiek	Commissie Medische Ethiek	Bruxelles Bruxelles<br>Belgium
9016	Commissie Medische Ethiek	Commissie Medische Ethiek	Bruxelles Bruxelles<br>Belgium
9017	Comite d'Ethique hospitalo-<br>facultaire	Comite d'Ethique hospitalo-facultaire	Luik Luik<br>Belgium
9020	Ethisch Comite Sint Jan	Ethisch Comite	Brugge West Vlaanderen<br>Belgium
9021	Ethisch Commissie GZA	Ethisch Commissie GZA	Wilrijk Antwerpen<br>Belgium
9022	Comite d'Ethique	Comite d'Ethique	Charleroi<br>Belgium
9023	Comite d'Ethique	Comite d'Ethique	Bruxelles<br>Belgium
9024	Comite d'Ethique	Comite d'Ethique	Yvoir<br>Belgium
9025	Ethisch comite UZ Gent	Ethisch comite UZ Gent	Gent<br>Belgium

9026	Commissie voor Medische Ethiek	Commissie voor medische ethiek	Kortrijk<br>Belgium
9027	CRP sante	Comite d'Ethique	Luxembourg<br>Luxembourg
